# Modeling, applications and challenges of inner ear organoid

**DOI:** 10.1002/SMMD.20230028

**Published:** 2024-01-16

**Authors:** Jieyu Qi, Liyan Zhang, Xiaohan Wang, Xin Chen, Yiyuan Li, Tian Wang, Peina Wu, Renjie Chai

**Affiliations:** ^1^ State Key Laboratory of Digital Medical Engineering Department of Otolaryngology Head and Neck Surgery Zhongda Hospital School of Life Sciences and Technology Advanced Institute for Life and Health Jiangsu Province High‐Tech Key Laboratory for Bio‐Medical Research Southeast University Nanjing China; ^2^ Co‐Innovation Center of Neuroregeneration Nantong University Nantong China; ^3^ School of Life Science Beijing Institute of Technology Beijing China; ^4^ Department of Otolaryngology‐Head and Neck Surgery Stanford University School of Medicine Stanford California USA; ^5^ Department of Otolaryngology‐Head and Neck Surgery The Second Xiangya Hospital Central South University Changsha Hunan Province China; ^6^ School of Medicine South China University of Technology Guangzhou China; ^7^ Department of Otolaryngology Guangdong Provincial People's Hospital (Guangdong Academy of Medical Sciences) Southern Medical University Guangzhou China; ^8^ Department of Otolaryngology Head and Neck Surgery Sichuan Provincial People's Hospital University of Electronic Science and Technology of China Chengdu China

**Keywords:** hair cell, inner ear, organoid, stem cell

## Abstract

More than 6% of the world's population is suffering from hearing loss and balance disorders. The inner ear is the organ that senses sound and balance. Although inner ear disorders are common, there are limited ways to intervene and restore its sensory and balance functions. The development and establishment of biologically therapeutic interventions for auditory disorders require clarification of the basics of signaling pathways that control inner ear development and the establishment of endogenous or exogenous cell‐based therapeutic methods. In vitro models of the inner ear, such as organoid systems, can help identify new protective or regenerative drugs, develop new gene therapies, and be considered as potential tools for future clinical applications. Advances in stem cell technology and organoid culture offer unique opportunities for modeling inner ear diseases and developing personalized therapies for hearing loss. Here, we review and discuss the mechanisms for the establishment and the potential applications of inner ear organoids.

## Graphical abstract

Advances in stem cell technology and organoid culture offer unique opportunities for modeling inner ear diseases and developing personalized therapies for hearing loss. Resources for the inner ear organoid construction are emphasized. Recent advancements in modeling technologies for inner ear organoids are presented. The application challenges of inner ear organoids in the field of hearing reconstruction are presented. Finally, the authors review and discuss the mechanisms for the establishment and the potential applications of inner ear organoids.

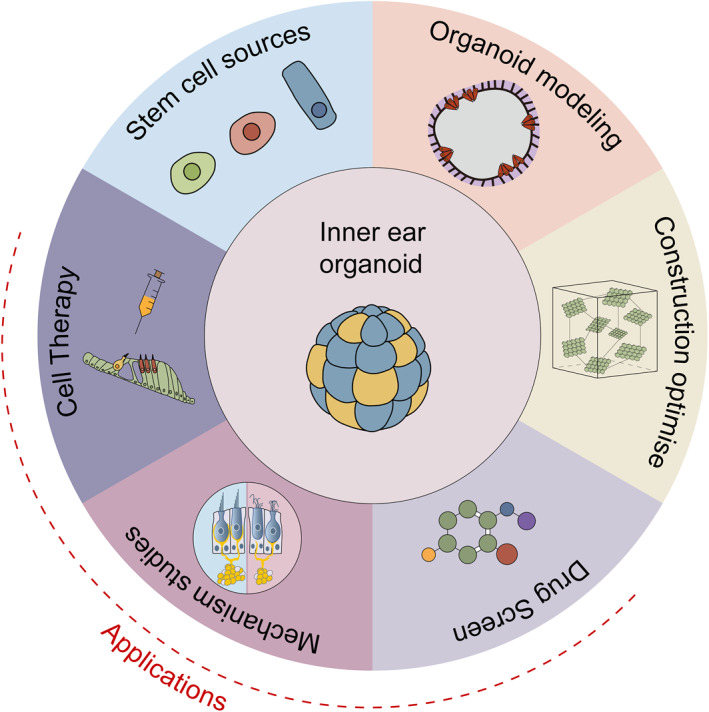

**Key points**

- The resources for the inner ear organoid construction are emphasized.
- Recent advancements in modeling technologies for inner ear organoids are presented.
- The application challenges of inner ear organoids in the field of hearing reconstruction are presented.

## 1 INTRODUCTION

In 2019, 1.57 billion people worldwide were affected by hearing loss, accounting for about one‐fifth of the total population. Hearing loss ranks among the top three causes of health‐impairing disabilities and injuries in the “Global Burden of Disease” analysis, along with osphyalgia and migraine. Most permanent hearing loss is sensorineural.^1^ The sensory organ in the inner ear contains the vestibular and auditory receptors. The mechanoreceptors in the inner ear consist mainly of hair cells and surrounding supporting cells.^2^ Hair cells can convert mechanical stimuli into electrochemical signals. Hair cells also reside in other sensory tissues in the vestibular organ, including the saccule, utricle and ampullae of the semicircular canals, which sense linear motion, gravity and head rotation to maintain balance.^3^ Two types of hair cells are classified in the cochlear sensory epithelium, named inner hair cells and outer hair cells, which act as the primary sound receptors by amplifying sound‐induced vibrations in the epithelium.^4^, ^5^ Hair cell damage can activate mitosis and differentiation of supporting cells.^6^, ^7^ New stem cell therapies in the cochlea are being developed along with gene and drug therapies for hearing loss treatments.

Stem cell‐derived organoids are an important tool for developing and validating cell therapies. Organoids refer to cell aggregates formed by self‐assembly of embryonic stem cells (ESCs), tissue progenitor cells and induced pluripotent stem cells (iPSCs) ex vivo,^8^ which can partially mimic the morphology, structure and function of cochlea tissue.^9^, ^10^ In 2009, Clevers et al. first successfully cultured real intestinal organoids in vitro,^11^ which opened a new chapter in organoid research. In recent decades, with the advancement of in vitro stem cell culture technology and a deeper understanding of extracellular matrix (ECM), organoid culture systems of different organ tissues have been valued, established and optimized gradually. At present, a variety of organoid culture technologies for the brain, kidney, lung, and intestine have been established.^12^ The rapid development of organoid technology plays an extremely crucial role in studying the mechanisms of deafness occurrence and development,^13^ establishing a new drug screening protocols^9^ and therapeutic strategies.^14^

**FIGURE 1:**
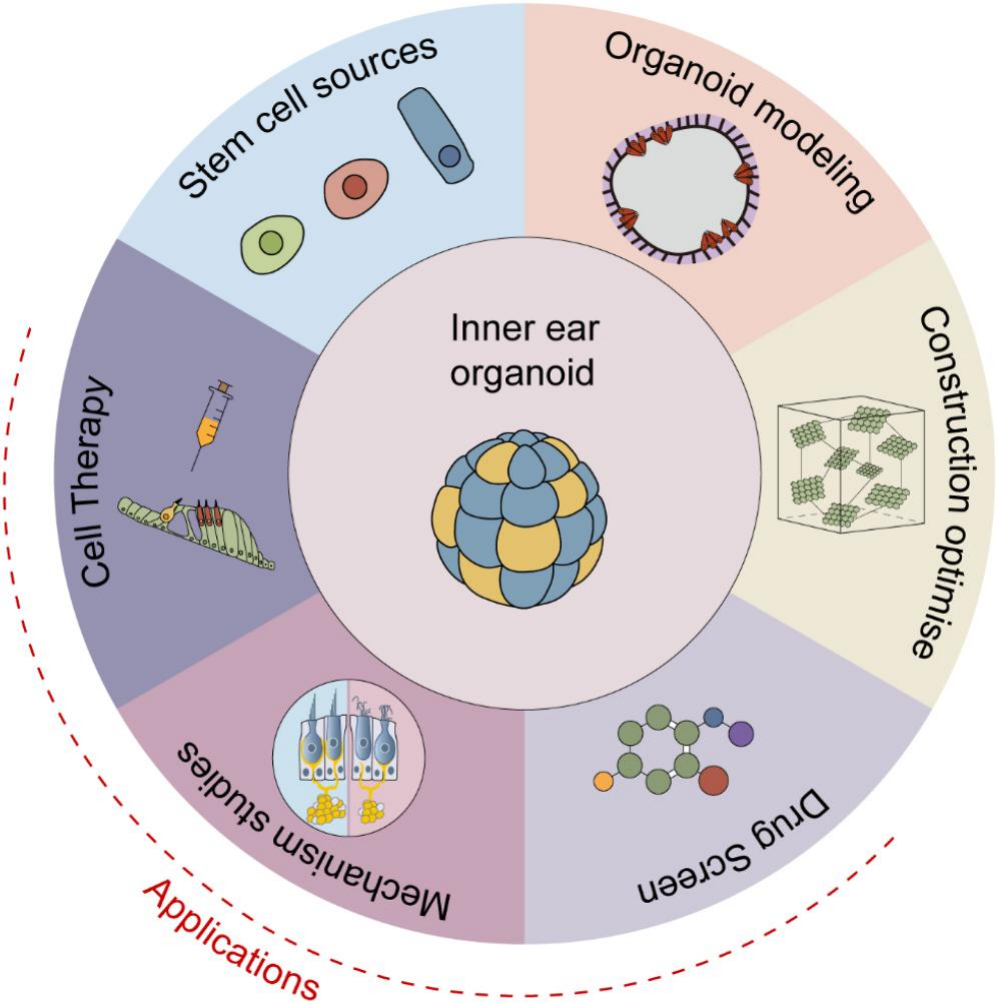
Schematic illustration of recent advancements in modeling and applications of inner ear organoids.

Most of the broad comprehension of the cell and molecular biology of the human auditory system and related hearing loss has been deduced from experimental studies and observations in animal or cell models. Because it is not possible to perform biopsies on living humans without generating major irreversible damage to the auditory or vestibular organs, studies of the human inner ear are typically limited to cadaveric tissues and scarce fetal. Developing organoids to model inner ear development and disease provides unprecedented opportunities to develop new therapeutic methods, such as stem cell therapies, while also providing excellent preclinical testing in high‐fidelity models. In this review, we discuss methods for constructing inner ear organoids, compare differences in organoids derived from different stem cells, summarize the advances and key challenges in inner ear organoid applications, and provide insights into the widespread application of stem cell therapy in the treatment and prevention of sensorineural hearing loss (Figure 1).

## 2 INNER EAR

**FIGURE 2:**
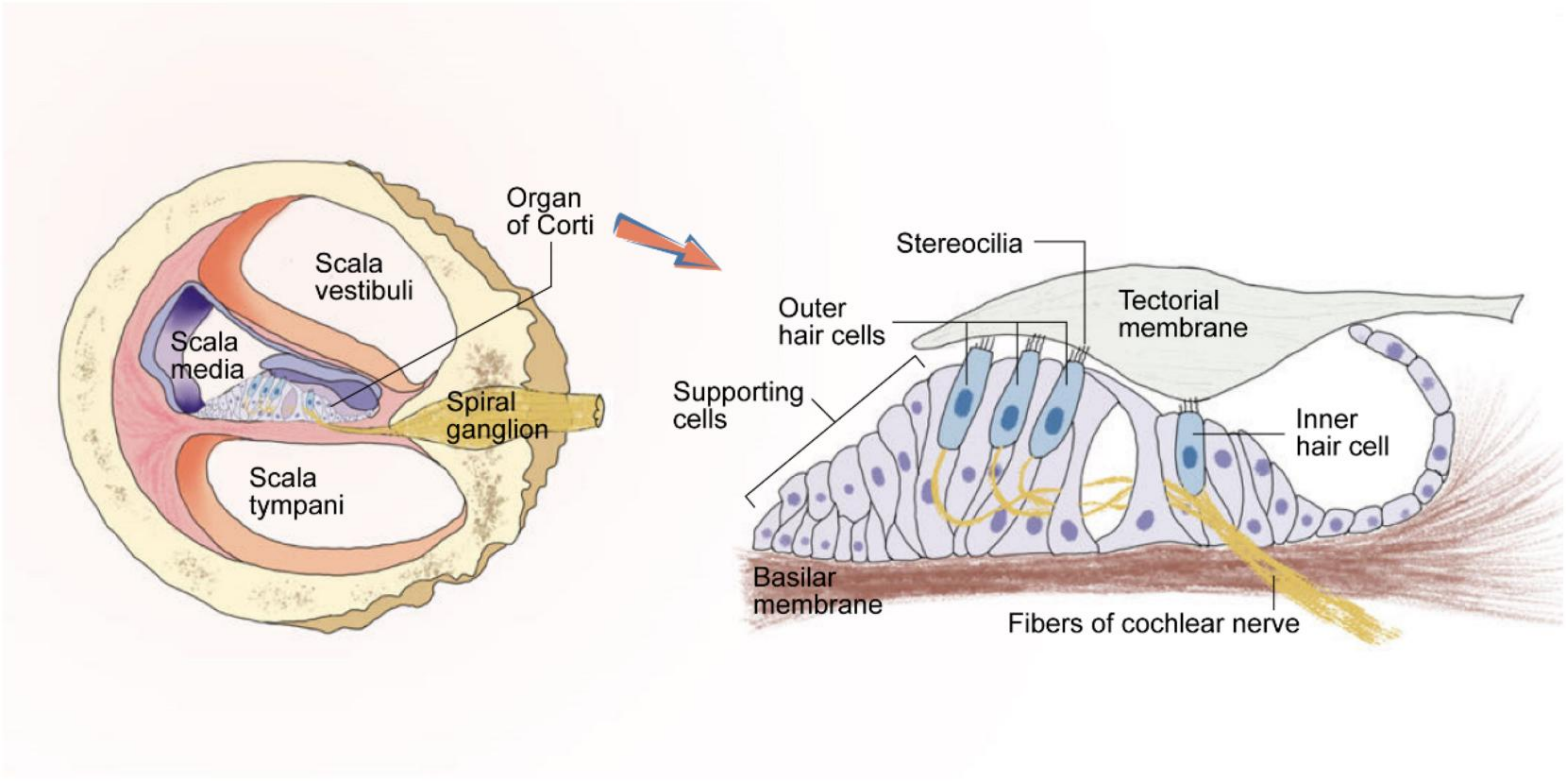
Schematic diagram of the anatomical relationships of the inner ear. Left: Composition of the cochlear duct. Right: Structure of the organ of Corti.

The auditory system is the perceptual system of hearing sensation and contains both peripheral and central components. The inner ear is located deep within the peripheral auditory system, wrapped around the temporal bone, and has a complex and sophisticated structure. The acoustic vibrations produced by sound are collected and reflected by the auricle, resonantly amplified by the external auditory canal, pressurized and amplified by the auditory ossicles, and finally transmitted from the oval window into the cochlea, which is responsible for sensing the hearing in the inner ear, named for its resemblance to a coiled spiral, and consists of a central modiolus and a surrounding bony cochlea. The spiral apparatus within the cochlea, known as the organ of Corti, is the main part of the cochlea that senses inputting sound signals, and it works in concert with the innervating auditory nerves to transmit sound vibration signals into the auditory cortex (Figure 2).

Hair cells are the main functional organizers of the epithelial tissues in the inner ear. The inner ear contains six sensory epithelial tissues: one in the organ of Corti in the cochlea, which dominates the transduction of acoustic signals, and five in the vestibular apparatus, which detects linear acceleration and angular velocity. Cochlear hair cells are encapsulated by supporting cells. The supporting cells can provide strong support for the hair cells, separating the hair cells from neighboring ones. The inner hair cells are arranged in rows along the cochlea, and the outer hair cells have three to five rows. All hair cells form synaptic connections with bipolar neurons located in the spiral ganglion of the modiolus. Hair cells usually have many cilia arranged in a form from high to short by height. The stereocilia occupy most of the apical surface of the hair cell, and the longest kinocilium is located at the distal lateral edge of the apical surface, which serves to guide the formation of the stereocilia. When sound enters the inner ear, it causes the basilar membrane to vibrate. The shear force between the basilar membrane and the tectorial membrane deflects the stereocilia bundles, causing the opening or closing of the mechanically gated ion channels, thus converting the intensity and frequency information of the sound into electrical signals, which further causes the cells to release neurotransmitters, completing the process of mechanical signal—electrical signal—chemical signal transduction.^15^

## 3 CONSTRUCTION OF INNER EAR ORGANOIDS

**FIGURE 3:**
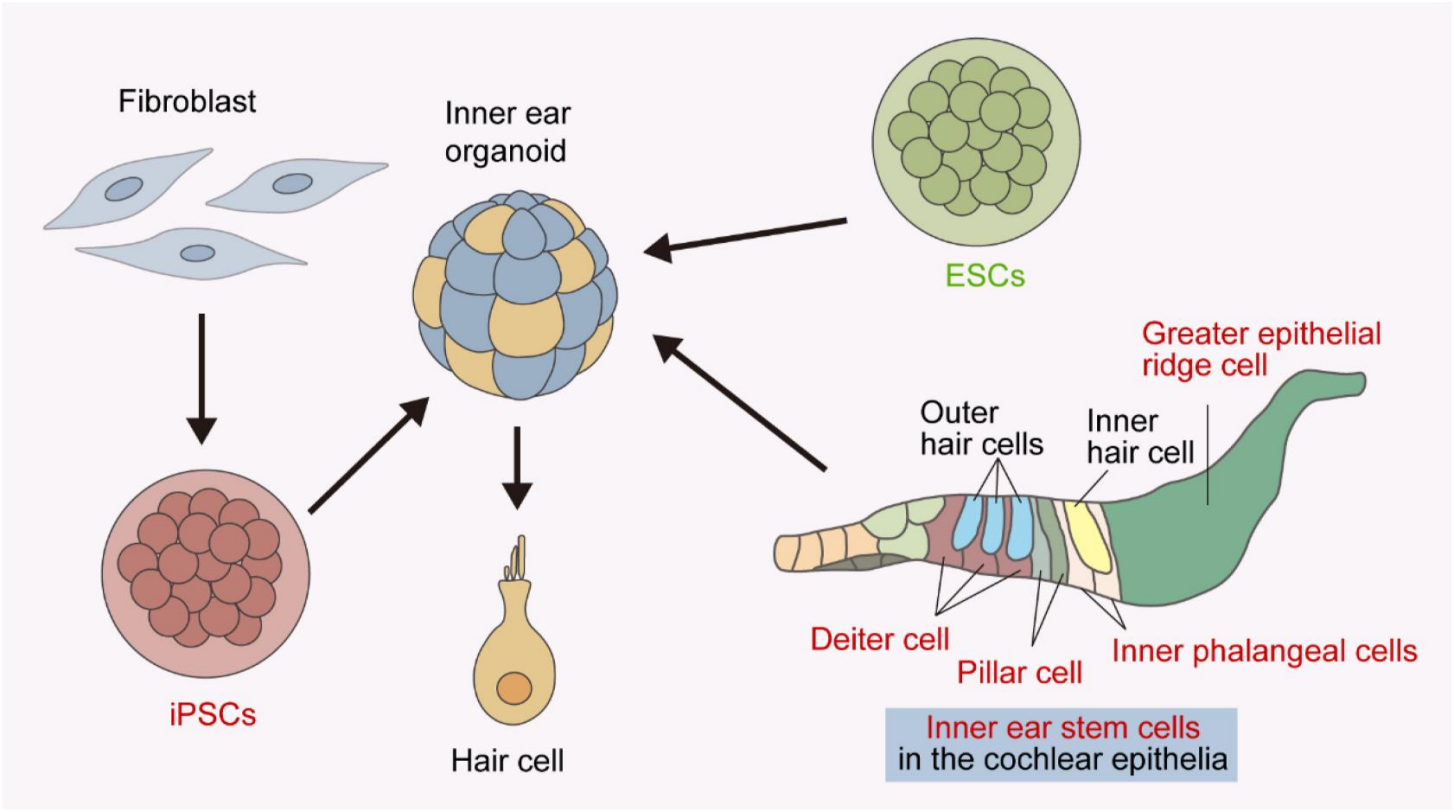
Origins of inner ear organoids. Inner ear organoids can be induced from either pluripotent or mono‐potent stem cells, including inner ear stem cells, ESCs, and iPSCs. ESCs, embryonic stem cells; iPSCs, induced pluripotent stem cells.

Inner ear organoids can be induced from pluripotent stem cells or mono‐potent stem cells, such as ESCs, iPSCs and inner ear stem cells (Figure 3). The formation of inner ear organoids from pluripotent stem cells requires a variety of cytokines or small molecule drugs to perfectly reproduce the various characteristics involved in the inner ear development, and adult stem cells can be directly induced to differentiate due to their mono‐potent properties.

### 3.1 Multifunctional stem cells

**FIGURE 4:**
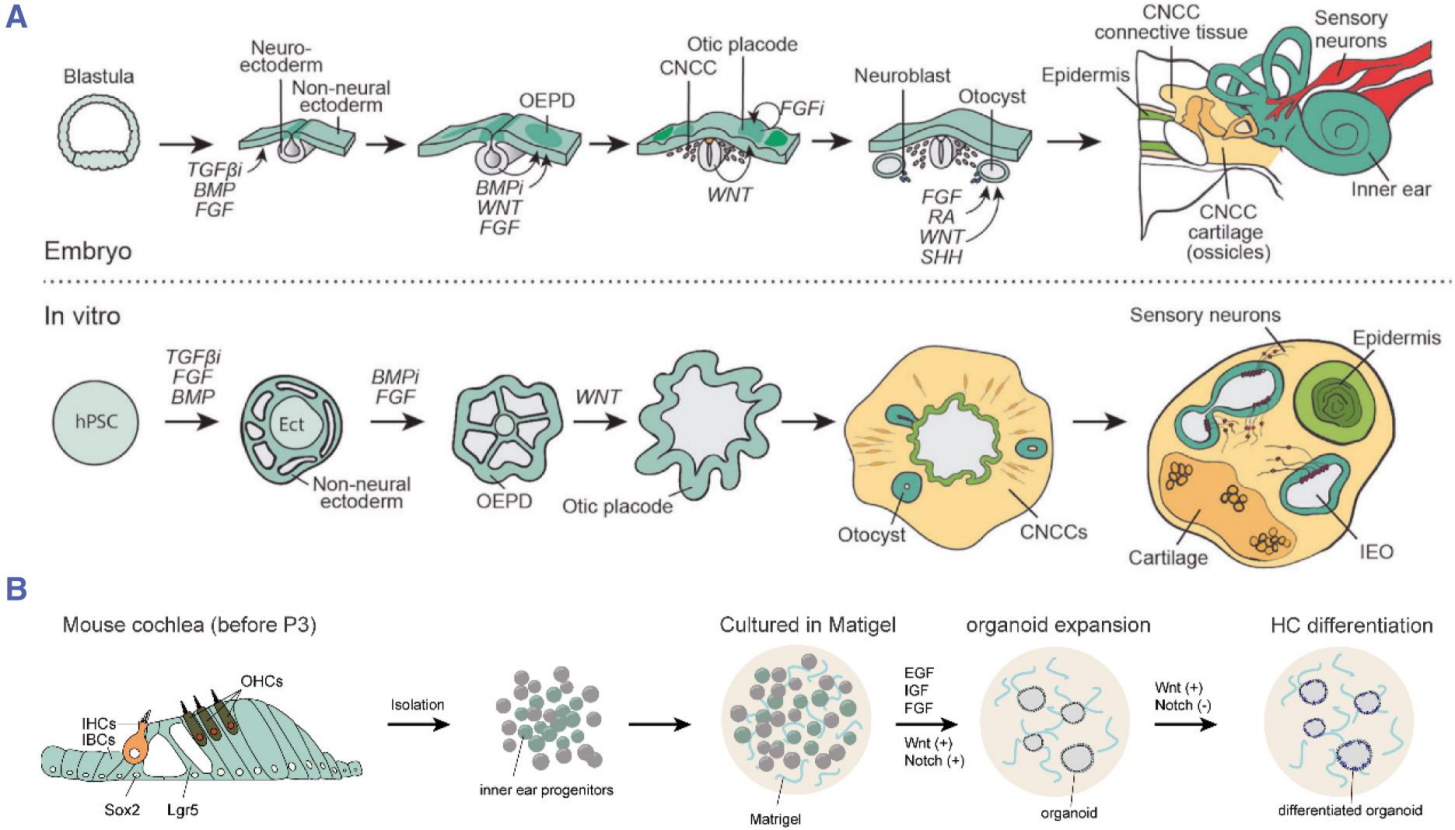
The construction of inner ear organoids. (A) Upper: The process of inner ear formation during the embryonic development. Lower: Schematic illustration of the procedure of organoid formation from pluripotent stem cells in a manner similar to that in the embryo. Reproduced under terms of the CC‐BY license.^16^ Copyright 2021, The Authors, published by Springer Nature. (B) Schematic illustration of the procedure of organoid formation from inner ear stem cells using Lgr5‐positive support as an example.

The inner ear originates from the ectoderm (Figure 4A). In human beings, the ectodermal epithelium is formed 12 days after fertilization. The epithelium splits into neural ectoderm and non‐neural ectoderm (NNE). The inner ear is eventually given rise from the NNE. The first hair cells with terminal differentiation are produced approximately 52 days after fertilization in humans. The site of inner ear genesis is derived from the auricular plate and is located in the intracranial junction zone between the NNE and the neural ectoderm, the otic‐epibranchial placode domain (OEPD).^17^, ^18^ The differentiation of human pluripotent stem cells to specific tissues requires mimicking the complex regulatory network of pathways and transcription factors during embryonic development (Figure 4A). Fate determination of inner ear organoids requires the precise regulation of WNT signaling, sonic hedgehog (SHH) signaling, transforming growth factor (TGF), fibroblast growth factor (FGF), bone morphogenetic protein (BMP), retinoic acid, and other signaling or factors. Cochlear epithelial progenitor cells differentiated from human ESCs form clusters of epithelial cells resembling hair‐cell‐like cells with ciliated bundles composed of actin co‐cultured with chicken ellipsoidal bursa stromal cells supplemented with epidermal growth factor (EGF) and retinoic acid.^19^ Chen et al. obtained differentiated inner ear progenitor cells cultured with N2, B27, FGF3 and FGF10 for 12 days using human ESCs cell line X1. The temporal expression patterns of Notch signals, a key signal in regulating hair cell differentiation, were detected and the possible co‐reregulated roles of Notch ligand JAG2 (jagged canonical Notch ligand 2) and DLL1 (delta‐like canonical Notch ligand 1) were hypothesized to be anticipated in the differentiation of hair cells.^20^ In another research, human ESCs (WA25 cell line) were incubated in an E8 medium containing low concentrations of Matrigel and FGF to induce the differentiation of the ectoderm to form dense spherical cell clusters. Afterward, the TGF signaling pathway was inhibited and ectodermal differentiation was stimulated with BMP‐4, resulting in the differentiation of NNE and neural ectoderm by day four. At the same time, the otic placode was concave inward to form an otocyst, and asymmetric gene expression led to the dorsoventral and anteroposterior differentiation. Subsequently, the inhibition of BMP signaling with LDN‐193189 and addition of FGF‐2 induced the differentiation of cell aggregates toward the OEPD, and the OEPD was formed on the eighth day, whereas subsequent treatment with CHIR99021, a glycogen synthase kinase 3β (GSK3β) inhibitor, revealed that the surface of the aggregates on the 12th day appeared as epithelial protrusions, predicting the formation of the inner ear organoids.^21^ Menendez et al. also induce hair cell‐like cells by adding a combination of four transcription factors (*Pou4f3*, *Atoh1*, *Gfi1*, and *Six1*) into the postnatal cochlear supporting cells, adult rat fibroblasts, and mouse embryonic fibroblasts with a higher transformation efficiency than *Atoh1* alone, a determinant for hair cell differentiation.^22^

iPSCs are another type of multifunctional stem cells, which are a class of pluripotent stem cells with properties similar to ESCs produced by adult somatic cells in response to some transcription factors. Takahashi et al. introduced a four‐transcription‐factor combination (including *Klf4*, *c‐Myc*, *Sox2*, and *Oct3/4*) into mouse fibroblasts and found that the fibroblasts were gradually transformed and gave rise to cells with characteristics similar to ESCs, including morphology, gene expression, cell proliferation and differentiation abilities.^23^ The process from iPSCs to inner ear organoids also requires multiple signaling pathways. So far, almost all studies of iPSCs on inducing inner ear organogenesis have been improved based on the step‐by‐step induction method conducted by Koehler et al.^1^, ^24^ Most of the induced hair cells exhibit characteristics of type II utricular hair cells, and the steps of induction mainly include (1) formation of the epidermal ectoderm and otic placode, (2) formation of the inner ear progenitors, and (3) formation of the fine cavity structure with sensory epithelium.^25^ Compared with ESCs, iPSCs have similar differentiation pluripotency and self‐renewal ability, while in terms of source, iPSCs can be generated through in vitro induction of somatic cells, avoiding the mobilization of embryos, thus reducing the controversy of medical ethics. Meanwhile, iPSCs are easily accessible and can be cloned in large numbers, providing a rich source of cells for in vitro stem cell research and cell therapy. However, there are certain problems with the use of iPSCs. The spontaneous differentiation and accumulation of chromosomal aberrations may impose limitations on iPSCs during passaging. In vitro culture of iPSCs also suffers from slow generation, inefficient induction, and poor uniformity.

### 3.2 Inner ear stem cells

The establishment of an in vitro organoid culture method has facilitated the isolation of progenitors residing in the cochlear tissue. The source of the clone‐forming ability and self‐renewal capacity of inner ear progenitor cells have been investigated. Researchers have found the presence of hair cell progenitors in the cochlear sensory epithelium that can be differentiated in vitro into either hair cells or supporting cells (Figure 4B).

Diverse subtypes of inner ear stem cells with regenerative potential have been identified. Cell sorting based on the expression of *Lgr5*, *Sox2*, *p27*, et al. was performed to isolate cochlear supporting cells, and the sphere culture results showed that several cell subtypes of the supporting cells had proliferative capacity.^7^, ^26^ Genes in the WNT signaling pathway have been recognized as markers of stem cells for a variety of adult tissues with strong proliferative and differentiated capacities.^27^ WNT signaling is necessary for cochlear development, with *Lgr5* being recognized as one of the most stringent stem cell marker genes for mouse cochlear progenitor cells.^7^, ^28^ *Lgr5* has been detected in the third row of Deiters cells, inner phalangeal cells, inner pillar cells, and inner border cells of supporting cells.^29^, ^30^ At the embryonic age of 18.5 days in mice, *Lgr5* is expressed in both hair cells and supporting cells, which is gradually downregulated thereafter.^29^ Studies have elucidated two mechanisms for the endogenous regeneration of cochlear hair cells: direct transformation of the supporting cells to hair cells, and mitotic division of the supporting cells to produce daughter cells that are subsequently reprogrammed into hair cells.^31^, ^32^ The Lgr5‐positive cells in the sensory epithelia of neonatal mice are reprogrammed and transdifferentiated into hair cell‐like cells in response to injury.^33^ The regenerative capacity of Lgr5‐positive cells can be strengthened by activating WNT signaling or inhibiting Notch signaling.^7^, ^28^, ^33^ The subpopulation of supporting cells expressed by other genes of the WNT pathway such as *Axin2*, *Frizzled9*, and *Lgr6* are also inner ear stem cells, all of which are capable of serving as a source of hair cell regeneration.^29^, ^34^, ^35^, ^36^, ^37^

In addition to the stem cell subtypes mentioned above, some cells located in the greater epithelial ridge (GER) region of the cochlea have the ability to regenerate Myo7A‐positive hair cells.^38^ These cells seemed to have the similar regeneration capacity in vitro as classical WNT‐positive inner ear stem cells. Traditionally, in situ hair cell regeneration has been considered critical for therapeutic research. Although stem cells in the GER region can regenerate hair cells, they are of little significance for in situ regeneration of hair cells in vivo. However, it should also be noted that GER's inner ear stem cells expand the cell source for the construction of inner ear organoids, provide more clues to the occurrence of inner ear lineages, and play a crucial role in in vitro screening for new regulatory factors.

Inner ear organoids can be induced from ESCs, iPSCs and mouse inner ear stem cells in the presence of specific ECMs (usually Matrigel) and cytokines, including Wnt signaling activators, growth factors such as EGF, FGF, BMP, B27, small molecule inhibitors, and related hormones that permit organoid growth.^39^ EGF, insulin‐like growth factor I (IGF‐1), and β‐FGF are thought to be involved in inner ear development due to their mitogenic function, the ability to promote survival activity, or induce certain cellular phenotypes. ESCs, due to their high totipotency, can be guided to the generation of a specific cell type through the application of cues in normal development. The differentiation of ESCs can be induced by the development of aggregated embryoid bodies in vitro. Embryoid‐derived cells were fate‐determined to inner ear progenitors in serum‐free culture medium with N2 supplementation, EGF, and IGF‐1, and further expanded using β‐FGF; subsequent removal of these growth factors‐initiated organoid differentiation, and the terminally differentiated hair cell markers were then strongly expressed and observed, like MYOSIN7A, ESPIN, and PARVALBUMIN et al. However, the newborn hair cells did not have the typical morphology and structure of native hair cells.^40^ In subsequent studies, through precise temporal control of BMP, TGF‐β, and FGF signaling, ESCs aggregated embryoid bodies are sequentially differentiated and the regenerated hair cells exhibited the similar functional mechanosensitivity of native hair cells and innervated with sensory neurons derived from ESCs cultures.^24^, ^41^ The inner ear organoid can also be induced by the addition of LY411575, the γ‐secretase inhibitor,^42^, ^43^ to a culture system containing the growth factors EGF, β‐FGF and IGF‐1, GSK3β inhibitor CHIR99021 and valproic acid (VPA, the histone deacetylase inhibitor) for the *Lgr5*‐positive stem cells derived organoids expansion.^44^, ^45^

Spiral ganglion neurons (SGNs), the primary auditory receptors, are bipolar neurons that connect hair cells and the auditory brainstem. During cochlear development, sensory hair cell precursors through terminal mitosis and subsequent differentiation are induced by the signals from neighboring SGNs.^46^ Co‐culture of inner ear organoids and neurons achieves the maturation of the cochlear organoids and the functionalization of newly formed hair cells.^47^, ^48^ In addition, functional synapses are established, which are crucial for the perception of mechanical signals.^48^ The generation of cochlear organoid with functional synapses in organoids and neurons co‐culture system helps us to understand how to re‐organize the hair cell synaptic machinery with similar morphology, physiology and molecular features of the native ones.

### 3.3 Construction of inner ear organoids

Given that the normal development of organs requires coordinated cell populations to accurately proliferate and differentiate, 2D cultures are defective in that they do not possess the complexity of tissue structure and cannot mimic the in vivo microenvironment in which cells are adhered to a culture plate or suspended in culture. To make the cell aggregates cultured in vitro more organized, 3D (three‐dimensional) culture technology has emerged. Totipotent stem cells are induced to develop into self‐organizing organoid structures by applying specific growth factors and small molecules in a 3D culture matrix. Cell self‐organization in a 3D matrix more closely mimics the self‐organization process during in vivo development, and its function is similar to that of a replicating organoid. 3D cultured organoids have been widely used in the induction of functional tissues, disease modeling, and drug screening, and have great potential for basic research and translational applications.^9^

Under 3D culture conditions, to optimize the cell‐cell and cell‐ECM connectivity in culture, in addition to inducing the cell development process through cytokines and small molecules, a variety of platforms for the optimization of 3D cell culture systems, which are mainly classified into two main types: scaffold‐less systems mediated by external forces (e.g., suspended droplet, rotary, and magnetic method) and 3D scaffold‐based systems (e.g., microfluidic chip and hydrogel matrix, etc.). The drawback of scaffold‐less systems is the inability to reproduce intercellular cell adhesion and cell migration, not to mention the inability to achieve the study of self‐organized systems. 3D scaffold systems, on the other hand, can adjust the degree of porosity, surface chemistry, and stiffness by adjusting the biological components as well as the synthetic materials to more accurately mimic the specific cellular tissue proliferation and differentiation, and the following self‐organization of the microenvironment, to achieve optimal conditions for organoid formation.^49^ Matrigel is currently used as a common scaffold system for 3D organoid culture, and the main components of this matrix are ECM proteins, including laminin, collagen IV and endomucin at the ratio of 60%, 30%, and 8%, respectively as well as several growth factors, including vascular endothelial growth factor, TGF‐β and IGF‐1. Isolated inner ear stem cells can be efficiently expanded and differentiated toward inner ear organoids under Matrigel‐based culture conditions.^50^

It has been reported that Matrigel‐based hydrogel matrices tends to cause poor experimental reproducibility due to the varying biochemical properties and growth factor concentrations in different batches. In addition, because it cannot be easily customized, it cannot be fully matched to the culture of different tissue‐like organoids. These undesirable properties of matrix gels have led to the investigation of other chemically and mechanically defined natural and synthetic scaffolds for organoid culture.^47^ Recently, the incorporation of Ti3C2Tx MXene nanomaterials and Matrigel modulated the identities of Matrigel, promoted cochlear organoid development, favored the hair cell formation inside, and facilitated the synapse formation efficiency in the organoid and SGN co‐culture system,^47^ furthering the application of biomaterials in research on inner ear organoid and hearing loss treatment. In the study of inner ear organoids, other biosynthetic scaffolds besides Matrigel have not been tested and applied. Better investigation of other extracellular scaffolds (natural and synthetic) to facilitate the reorganization of inner ear organoids into functional tissues is key to the development and improvement of inner ear organoids for regenerative medicine applications. For example, a 3D culture system for the cultivation of intestinal stem cells (ISC) and formation of intestinal organoids through polyethylene glycol hydrogels was used to create a 3D culture system by adding Arg‐Gly‐Asp peptide for ISC proliferation and laminin for organoid formation. Through this, a stable culture system was created for intestinal organoids by adjusting the physicochemical parameters of the system to achieve the optimal conditions for ISC expansion.^51^ Gelatin methacrylate (GelMA) hydrogels are also suitable for organoid culture studies due to their suitable biological properties and tunable physical properties, and hybrid hydrogel systems can also be used to form matrix networks with the corresponding combination of properties required for specific tissues by mixing GelMA with nanoparticles and other polymers.^52^ GelMA hydrogel has been applied to delayed‐release dexamethasone through intratympanic injection for the protection of noise‐induced hearing loss,^53^ but GelMA‐based organoid amplification studies have not been reported.

So far, 3D culture conditions still lack multi‐scale structural and tissue interfaces that make sense for organ function. In addition, the lack of control and precise application of nutrient gradients and chemical cues leads to poor modeling of the physiological microenvironment mimicking that in vivo. Also, cells are not exposed to physical stimuli that are essential for organ development and function. Although some 3D scaffolds that could mimic physical stimuli in situ have been used for organoid construction, these scaffolds have not been widely used due to their uncontrollable characteristics. Currently, organ‐on‐a‐chip is widely used to understand the molecular and cellular basis of various physiological and pathophysiological processes by simulating the physiologically complex organ systems in vitro, which is constructed through the miniaturized microchip manufacturing to accurately control the biophysical, biochemical and cellular parameters. Researchers have developed single organ‐on‐a‐chip for almost all important organs in the human body. In 2004, the first application of microfluidic technology to organ‐and system‐level l modeling with function for the study of human physiology or disease was published.^54^ The lung on a chip, known as the  “breathing lung,”^55^ initiated the development of organ‐on‐a‐chip, including multiple organs, such as the heart, liver, gut, and skin.^56^ The structure of the cochlea is very complex, including the bone labyrinth, membranous labyrinth, cochlear nerve and many other structures. Researchers have not yet summarized the clues of cochlear structural development to guide the development of mature cochlea in vitro, so the study of cochlear organoids is still in the preliminary stage. There have been some efforts to construct the cochlear organoids with partial function,^57^ but no studies on the inner ear‐on‐a‐chip have been reported.

## 4 APPLICATION OF INNER EAR ORGANOIDS

Inner ear organoids can be used to mimic the formation of cochlear hair cells. With similar morphology and function as normal cochlear hair cells, inner ear organoids are widely used for targeted drug screening and discovery, construction of disease models and investigation of corresponding mechanisms, hair cell regenerative medicine and organ repair.

### 4.1 Small molecule drug screening

High‐throughput drug screening for ototoxic, protective, or regenerative compounds is usually performed in vitro. Inner ear organoids are useful tools for studying drug toxicity and hair cell regeneration. McLean et al. screened a combination of small molecule compounds with high efficiency to induce hair cell regeneration by using organoids.^42^ CHIR99021 and VPA significantly promoted the expansion of inner ear organoids; 2‐phospho‐L‐ascorbic acid (pVc, a stabilized form of vitamin C) and the inhibitor of the TGF‐β receptor ALK5, named 616452, was able to further enhance the expansion effect of Lgr5‐positive supporting cells. The culture of inner ear organoids also requires many other growth factors, with bFGF and CHIR99021 being the most critical for Lgr5‐positive stem cell culture. The introduction of LY411575 enabled the gradual differentiation of organoids toward the hair cell fate. ERBB2, an EGF receptor, is known as a regulator of the cell cycle. Cochlear supporting cells respond to the activation of the ERBB pathway, leading to the supporting cells proliferation and hair cells differentiation.^58^ In inner ear organoids, the ERBB3‐binding protein inhibitors WS3 and WS6, in combination with CHIR99021, significantly promoted the formation of Lgr5‐positive inner ear organoids but did not affect hair cell differentiation.^59^ BIX01294, a G9a inhibitor, inhibited the proliferation of inner ear organoids in combination with CHIR99021.^59^ However, BIX01294 enhanced hair cell differentiation combined with a mixture of LY411575 and CHIR99021 in organoid medium.^59^ To discover new signals promoting hair cell differentiation, Liu et al. screened 1004 small molecules approved by the FDA using inner ear organoids from *Pou4f3* ^*EGF/+*^ mice in the presence of CHIR99021without LY411575, in which hair cells labeled with *Pou4f3*‐driven EGF were considered as the newly differentiated. The high‐throughput screen identified 91 candidate molecules that promote hair cell differentiation, of which regorafenib has good reproducibility at concentrations of 2, 5, and 10 mM. 5 mM regorafenib shows comparable differentiation ability in the hair cell production with LY411575, but the 15 mM regorafenib exhibits otologic toxicity.^60^ The establishment of a high‐throughput screening platform using a cochlear organoid is significant in identifying new small molecule drug candidates for hair cell regeneration and drug screening.

McLean et al. successfully compared the effectiveness of gamma‐secretase inhibitor (GSI) in hair cell regeneration experiments using a cochlear organoid model.^61^ The ototoxicity tests were then performed on rat cochlear organoids by 500 μM sisomycin exposure. Cochlear organoids treated with sisomycin showed obvious signs of apoptosis in the hair cell subpopulation. The sensitivity of the cochlear‐like organoids to sisomycin also verified the hair cell differentiation with function. Importantly, the author also verified that CPD3, a novel GSI, can replenish the number of hair cells lost in the ototoxicity test.^62^ It is important to note that the organoid culture model has limitations in drug screening. Since the medium components have been selected to maximize stem cell characteristics, the results obtained under in vitro conditions may not accurately reflect the changes obtained from a single drug in an intact organ in vivo. In addition, it needs to be noted that because the isolated and pooled supporting cells from the cochlea are cultured under homogenous conditions, precise information on cell type and anatomical structure is not available for cochlear organoids for cells that undergo damage or regeneration. Organoids can also be used to build organ chips for biomedical research and drug development.^63^, ^64^

### 4.2 Deafness modeling and mechanism studies

Inner ear organoids provide a research model for the study of hearing loss caused by genetic defects. Mutations in TMPRSS3, a type II transmembrane serine protease, cause congenital and early‐onset hearing loss. Inner ear organoids containing the TMPRSS3 mutation (TMPRSS3^Y260X^) were able to undergo organoid expansion and hair cell differentiation, but degeneration and apoptosis of hair cells were detected. On day 38 of culture, the big potassium ion channels of hair cells were reduced in *Tmprss3* knockout organoids, which was consistent with that of mouse vestibular hair cells, suggesting that mouse *Tmprss3* knockdown inner ear organoids can mimic the pathological features of cochleas associated with these gene mutations. Single‐cell RNA sequencing results of *Tmprss3‐*knockdown inner ear organoids suggest that this organoid can be used as a tool to explore the molecular mechanisms of hair cell degeneration induced by *Tmprss3* knockdown.^65^ Hosoya and colleagues extracted iPSCs from Pendred syndrome patients with mutations at the double allele *SLA26A4* locus to establish an inner ear organoid model,^66^ revealing the molecular mechanism of PENDRIN dysfunction‐related hearing loss.

Mutations in *GJB2* (gap junction beta 2, encoding gap junction protein 26 [Connexin26, CX26]) cause severe human non‐syndromic sensorineural hearing loss. Fukunaga et al. obtained the CX26‐expressing cells and formed a typical CX26 gap junction plaque (CX26‐GJP) similar to that in the cochlea by mouse iPSCs.^67^ These CX26‐expressing cells induced by iPSCs exhibited spontaneous Ca^2+^ transients. This Ca^2+^ activity required the involvement of ATP and hemichannels, which is consistent with that in the normal developing cochlea. In addition, differentiated cells from iPSCs of CX26‐deficient mice also had the potential to form GJPs with dramatically fragmented small vesicle‐like GJPs, which is a major pathological feature of GJB2‐associated hearing loss. This in vitro organoid model may facilitate drug screening and cell therapy for GJB2‐associated hearing loss.

iPSCs‐derived inner ear‐like organ models have also been used to study the pathology of hearing loss, and test the gene correction efficiency, etc. Chen et al. obtained fibroblasts from patients harboring *Myo7a* and *Myo15a* mutations and the induced hair cells exhibited abnormal stereocilia. The disrupted stereocilia‐like protrusions were restored after gene correction with CRISPR/Cas9 technology.^68^ Together, the ability of inner ear organoids containing specific mutations to reproduce genetically relevant phenotypes observed in vivo suggests that organoid systems can serve as useful tools for understanding the genetic basis of hearing loss. The inner ear organoid provides a convenient in vitro disease model and provides a practical basis for the investigation of relevant mechanisms and therapeutic approaches in the field of the inner ear.

### 4.3 Hair cell regeneration therapy

**FIGURE 5:**
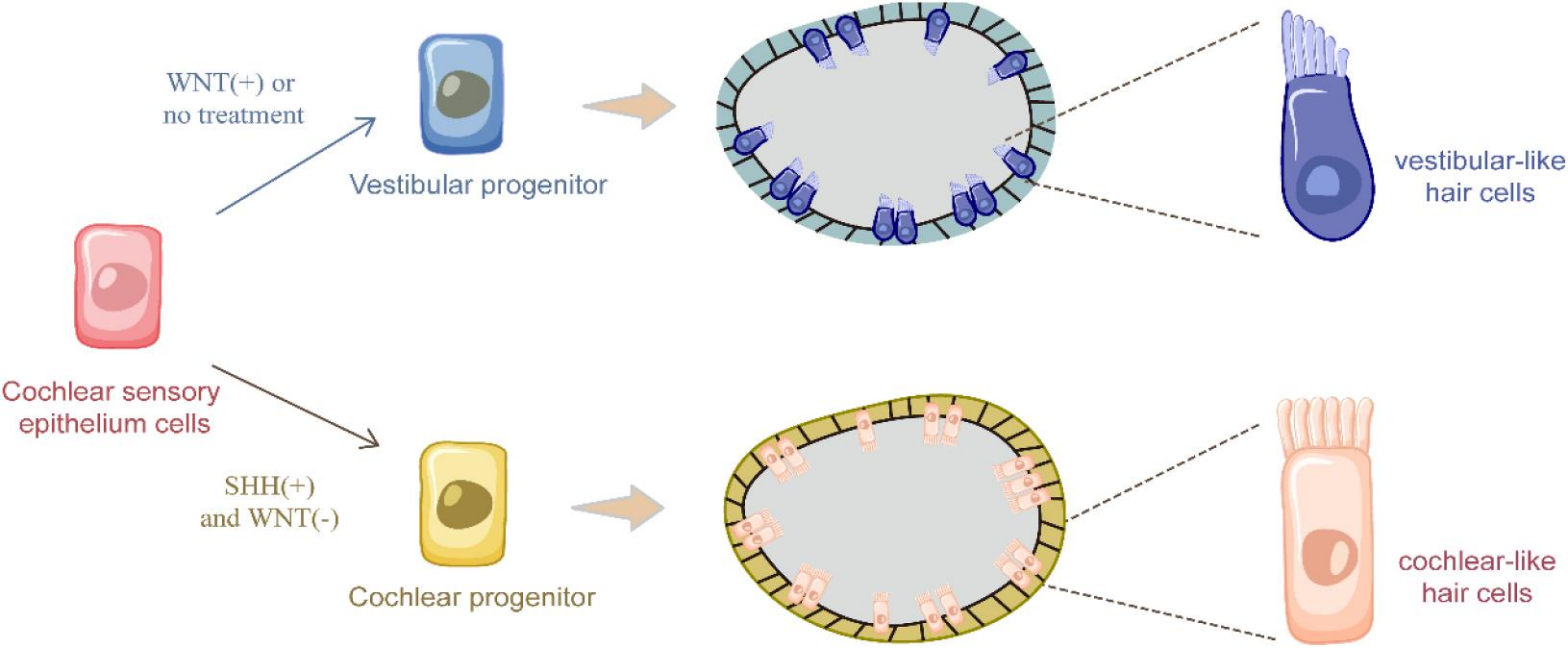
Schematic illustration of the process of cochlear organoid formation from human pluripotent stem cells. Simulating inner ear development, timed modulations of SHH and WNT signals results in the production of both outer and inner hair cell‐like cells in the cochlear organoids. SHH, sonic hedgehog.

The key scientific issue in stem cell‐based regeneration therapy in the cochlea is the regeneration of hair cells with physiological function. Studies have yielded differentiated hair cells of varying maturity. The differentiation efficiency and maturity of terminally differentiated cells remain very limited. Usually, growth factors or small molecules need to be added during the culture process to regulate the plasticity of cochlear progenitors and fate decisions. Koehler et al. succeeded in obtaining vesicular structures containing functional sensory hair cells from mouse ESCs with a patchy distribution of differentiated hair cells, similar to vestibular equilibrium sensory organs.^23^ They have also demonstrated that the hair cells of the ear‐like organ regenerated by ESCs are closer to vestibular hair cells than to cochlear hair cells in their electrophysiological properties, the morphology of the ciliary bundles and synaptic connections.^23^ Van der Valk et al. provided a detailed overview of the cell types represented in the differentiated growing organoids as well as the missing cell types. Analysis of most scRNA‐seq datasets suggests that the hair cells produced in ear‐like organs are essentially vestibular, but some speculative reports suggest that putative cochlear cells have been produced.^16^ In addition, some studies may indicate that the current culture process for cochlear organoids lacks growth factors that differentiate toward the cochlear fate. SHH signaling is thought to induce homogeneity in the ventral part of the otocyst.^69^ By replicating key differentiation cues in cochlea development, Moore et al. optimized the cochlear organoid induction protocol by sequentially modulating the SHH and WNT signaling pathways, leading to the development of the cochlear organoids with outer and inner hair cell morphology and marker expression, structural features, and electrophysiological characteristics.^57^ We summarized the formation process of vestibular and cochlear hair cells in the cultured organoid (Figure 5). In addition, ESCs cultured by Perny et al. also produce bipolar neurons, which are capable of forming synaptic connections with nascent hair cells.^70^ However, due to the large heterogeneity between ESCs or iPSCs‐derived inner ear progenitor cells and hair cells and those of the auditory epithelium, this greatly limits the application of ESCs or iPSCs‐derived inner ear cells in research.

Human tissue‐specific cochlear progenitor cells can also be obtained from the human embryonic cochlea. Roccio et al. found that these cochlear progenitor cells also express *p27*, *Sox2*, *Lgr5*, and *p75*.^71^ Cultured in Matrigel, these mitotically quiescent progenitor cells, stimulated by growth factors, were able to re‐enter the mitotic cycle, differentiate and exhibit sensory epithelial organization and polarity. These organoids were able to form organoids containing supporting cells and hair cells with an ESPIN‐ and F‐actin‐positive ciliary bundle after several weeks. Ototoxic drugs such as aminoglycoside antibiotics such as gentamicin, were able to produce toxicity to the regenerated hair cells, suggesting that human embryonic cochlear organoids can be used for in vitro screening of ototoxic drugs. These tissue‐specific progenitor cells are also significantly limited in their ability to proliferate and differentiate, and features such as morphology and structure are still far from the real organs.

Transplantation of in vitro amplified organoids into animals to repair damaged organs is feasible. However, cochlear organoid repair in animal models is still at the stage of in vitro studies. Other types of organoids have been reported in animal models. Rossi et al. transplanted the optic epithelial organoids constructed using mouse ESCs or mouse iPSCs in a mouse model with retinal degeneration and successfully generated mature photoreceptors and established synaptic connections with host neurons, restoring the response to light.^72^ Shirai et al. demonstrated that optic epithelial‐like organs (derived from human ESCs) survived, differentiated, matured, and exhibited some degree of integration with host tissues after transplantation in rats and primates with retinal degeneration.^73^ Yui et al. repaired colonic mucosal injury to varying degrees by transplanting the mouse colonic epithelial cells or stem cell‐derived intestinal organs into mouse models.^74^ Similarly, Fordham et al. proved that the transplanted intestinal organoids obtained from fetal intestinal progenitors differentiated from the injured mouse colon.^75^ In addition, organoids are also capable of providing ecological niches that protect grafts from adverse pathologic environments. Assawachananont et al. showed that organoids integrate better into the retina than the isolated retinal progenitor cells from embryos.^76^ Although only preliminary in vitro results were obtained, these results suggest that it is feasible for the clinical transplantation of stem cell‐derived organoids or tissues.

Obstacles to transplant inner ear organoids arise primarily from the complex organization of the cochlea, such as the spiral cochlea et al. Growing an intact cochlea in a petri dish for transplantation is currently far from feasible. However, elucidating the genetic events during the morphological acquisition of the cochlea and simulating these procedures is essential for the acquisition of cochlear organs in petri dishes on a large scale. Ishii et al. demonstrated that the extracellular regulated protein kinases signaling pathway is essential for cochlear duct elongation and coiling, and may be a key component to activate the acquisition of a more structurally cochlear spiral.^77^ Hocevar et al.^34^ have also shown that the provision of extracellular component matrix is critical for the normal formation of cochlea‐like organs.^50^ Many cues for cochlear morphogenesis remain preliminary. Only some progress has been made in obtaining human inner ear epithelial cells. Continued insight into the precise spatiotemporal signaling patterns of the embryonic inner ear is essential for improved organoid culture and applications.

## 5 SUMMARY

Since it is delicate and complex, methods to develop organoids that can maximally mimic the inner ear in vitro have not yet been developed. Unlike monolayer culture models, cells receive different doses of signals based on the spatial position within the organoid, leading to asynchronous differentiation of cells. In addition, inner ear organoids only produce vestibular sensory‐like structures and unmatured cochlear hair cells. Further modifications to coordinate regulation strategies are needed to develop new methods for inducing and differentiating diverse cell types with isolated characters, including how to derive mature cochlear hair cells, whether newborn hair cells have intercellular connections, whether the vulnerability of inner ear organoids are similar to that of native cochlea in response to the same extrinsic damages that result deafness in humans and other mammals, and how to recapitulate the vascular system that regulates blood supply to the inner ear in vitro. In addition, the efficiency and reproducibility of organoid production should be improved due to the heterogeneity of inner ear organoids from different sources. There is still a big step to straddle to achieve the goal of producing functional inner ear organoids in culture dishes for clinical drug screening and stem cell therapies.

## AUTHOR CONTRIBUTIONS

Jieyu Qi and Liyan Zhang prepared the manuscript. Jieyu Qi and Xiaohan Wang prepared the figures. Xin Chen and Yiyuan Li helped with the draft. Tian Wang, Peina Wu, and Renjie Chai provided crucial review and supervised the manuscript.

## CONFLICT OF INTEREST STATEMENT

The authors declare that there are no conflicts of interest.
